# CCL2 and CXCL2 are markers of delirium vulnerability in community dwelling older people

**DOI:** 10.1093/ageing/afag290

**Published:** 2026-09-30

**Authors:** Hannah Moorey, Hannah F Botfield, Maria Krogseth, Geir Selbaek, Daisy Wilson, Torgeir Bruun Wyller, Thomas Andrew Jackson

**Affiliations:** Department of Inflammation and Ageing, University of Birmingham College of Medical and Dental Sciences, Birmingham, UK; Department of Inflammation and Ageing, University of Birmingham College of Medical and Dental Sciences, Birmingham, UK; Oslo Delirium Research Group, Department of Geriatric Medicine, Oslo University Hospital, Oslo, Norway; Old Age Psychiatry Research Network, Telemark Hospital Trust, Skien, Norway; Department of Medical Biochemistry, Vestfold Hospital Trust, Tønsberg, Norway; Norwegian National Centre for Ageing and Health, Vestfold Hospital Trust, Tønsberg, Norway; Department of Geriatric Medicine, Oslo University Hospital, Oslo, Norway; Institute of Clinical Medicine, University of Oslo, Oslo, Norway; Department of Inflammation and Ageing, University of Birmingham College of Medical and Dental Sciences, Birmingham, UK; Department of Geriatric Medicine, Oslo University Hospital, Oslo, Norway; Institute of Clinical Medicine, University of Oslo, Oslo, Norway; Department of Inflammation and Ageing, University of Birmingham College of Medical and Dental Sciences, Birmingham, UK

**Keywords:** delirium, biomarkers, CCL2, CXCL2, chemokines, older people

## Abstract

**Background:**

Delirium is common and age, frailty and dementia are risk-factors. The pathophysiology is complex and poorly understood, but age-related immune system changes may be key. This study aimed to measure markers of immune cell migration, accelerated immune ageing and blood–brain barrier permeability in community-dwelling, older people comparing those who developed ≥1 episode of delirium over 2 years with those that did not.

**Methods:**

Baseline samples were collected from CASCADE participants who were followed up for 2 years. Delirium screening was performed weekly; positive screens or hospital admission triggered a delirium assessment. Serum was isolated, stored and analysed using a multiplex panel comparing levels between participants with and without delirium episodes.

**Results:**

About 98 participants were included. Median age was 88 (IQR: 81.8–92.0), with 90.8% classified as frail. Forty-three participants (43.9%) experienced ≥1 delirium episodes. CCL2 (MCP-1) and CXCL2 (MIP-2α), measured in a stable state at baseline, were significantly higher in the delirium group compared to the no delirium group (13.38 (11.21–17.93) vs. 10.83 (9.03–14.64) *P* = .004) and (17.23 (11.80–24.22) vs. 13.49 (10.19–17.98) *P* = .031), respectively. When stratified by dementia status, the associations of CCL2 with delirium were maintained in both groups, but CXCL2 was only significantly higher in delirium in the subgroup without dementia.

**Discussion:**

CCL2 and CXCL2 are chemokines involved in immune cell recruitment and markers of accelerated immune ageing. Future work should elucidate the role of immune cell recruitment and brain infiltration in delirium, to identify treatment targets. Furthermore, work should explore whether targeting immune ageing can reduce delirium risk.

## Key points

- This is an important study population, with a high prevalence of frailty, that are often excluded from delirium research studies.
- CCL2 and CXCL2 were significantly higher at baseline in participants in the delirium group compared to the no delirium group.
- CCL2 and CXCL2 are chemokines involved in immune cell recruitment and are also markers of accelerated immune ageing.
- Future work should identify potential treatment targets and assess whether geroprotectors can reduce delirium risk.

## Background

Delirium is an acute neuropsychiatric condition characterised by changes in attention, cognition and awareness [1]. Delirium affects 20% of older people admitted to hospital [2, 3] and is associated with poor outcomes [4]. The pathophysiology is complex and not fully understood. However, common triggers, such as infection and surgery, may activate the peripheral immune system, leading to neuroinflammation, blood–brain barrier (BBB) dysfunction and neuronal injury [1]. Older age is a risk factor for delirium and the immune system changes with age [5]. Markers of accelerated immune ageing are one factor that may explain vulnerability to delirium. Most studies focus on surgical cohorts; community-based studies in frailer populations are needed to identify markers of vulnerability in high-risk groups. We investigated delirium vulnerability by measuring markers of: (i) immune cell activation, adhesion and migration, (ii) accelerated immune ageing and (iii) BBB dysfunction at baseline in older people with one or more delirium episodes over a 2-year follow-up period, compared to those with no episodes.

## Methods

### Study design and participants

This study used a subset of 98 participants from the CASCADE cohort [6], a prospective, 2-year follow-up cohort study of older people (≥65) living in Norway and receiving some degree of domiciliary care services. Participants were recruited May 2015–July 2016. All participants gave informed written consent unless they lacked capacity to do so, where proxies gave consent on their behalf. The Norwegian Regional Committees for Medical and Health Research Ethics (2014/1972) granted ethical approval.

### Clinical assessments

The research team undertook assessments at the time of study enrolment, including a frailty assessment (Frailty Index) (supplementary material, Appendix S5). Dementia was diagnosed by consensus according to International Classification of Disease 10 criteria. Once per week the domiciliary care team were asked the Single Question in Delirium. A positive screen or hospital admission triggered a full assessment. Delirium was diagnosed using Diagnostic and Statistical Manual of Mental Disorders, fifth edition (DSM-5) criteria. A blood sample was taken at the time of enrolment, serum isolated and stored at −80°C.

### Laboratory methods

Multiplex analysis was performed using a Human Luminex Discovery Assay Kit (R&D Systems, USA) as per manufacturer instructions (supplementary material, Appendix S3 and S4).

### Statistical analysis

Statistical analyses were performed using IBM SPSS Statistics 30 and GraphPad Prism 10. Normality was assessed using Shapiro–Wilk Test and review of the data. Parametric results are presented as mean (SD) and nonparametric as median (IQR). For parametric results, levels in each group were compared using an unpaired *t-*test and for nonparametric, a Mann–Whitney U test. A receiver operating curve (ROC) assessed prediction of delirium for any analytes with significant associations.

## Results

The median age was 88 years (IQR: 81.8–92.0), and participants were almost exclusively frail (90.8%). Forty-three participants (43.9%) had at least one episode of delirium during the 2-year follow-up period, and the median time to delirium was 302 days (IQR: 109–492). The delirium and no delirium groups were matched (Table 1), except more participants in the delirium group had dementia (69.77% vs. 36.36%, *P* = .001), and a higher mortality during the follow-up period (41.8% vs. 20%, *P* = .019). There were no differences between the subgroup and the full CASCADE sample regarding the baseline data (supplementary material, Appendix S2).

**Table 1:** Demographics for study sample and comparing those that had at least one episode of delirium and those that had no episodes during the 2-year follow-up period.

	Total population (*n* = 98)	No episodes of delirium (*n* = 55)	One or more episode of delirium (*n* = 43)	Significance
Age (median/IQR)	88.0 (81.8–92.0)	89.0 (82.0–92.0)	87.0 (81.0–92.0)	*P* = .494
Female (%)	64 (65.3%)	38 (69.0%)	29 (67.4%)	*P* = .862
Frailty index score (mean/SD)	0.38 (0.11)	0.37 (0.11)	0.40 (0.12)	*P* = .225
Frail (%)^a^	89 (90.8%)	50 (90.9%)	39 (90.7%)	*P* = .972
Barthel index (median/IQR)	17 (15–18)	17 (15–18)	17 (15–19)	*P* = .896
Totally dependent (%)	90 (91.8%)	52 (94.5%)	38 (88.4%)	*P* = .268
Age adjusted CCI (Median/IQR)	6 (5–8)	6 (5–8)	6 (5–8)	*P* = .860
Dementia (%)	50 (51.02%)	20 (36.36%)	30 (69.77%)	***P* = .001**
IQCODE >3.3 (%)^b^	54 (55.1%)	25 (45.5%)	29 (67.4%)	***P* = .030**
IQCODE (median/IQR)	3.38 (3.06–4.07)	3.19 (3.00–3.50)	3.72 (3.13–4.38)	***P* = .004**
MOCA (median/IQR)	18 (14–22)	19 (14.0–23)	18 (11–20)	*P* = .126
Education (years, median/IQR)	8 (7–12)	8 (7–11)	8 (7–13)	*P* = .302
Mortality within study period (%)	29 (29.6%)	11 (20.0%)	18 (41.8%)	***P* = .019**

One or more episode of delirium group compared with no delirium episodes group. Parametric values compared using independent *t*-test. Nonparametric values compared using Mann–Whitney U test. Significance set at 95%. SD, standard deviation, IQR, interquartile range, CCI, Charlson comorbidity index, IQCODE, Informant Questionnaire on Cognitive Decline in the Elderly, MOCA, Montreal cognitive assessment.

^a^FI cut-off value ≥0.25.

^b^IQCODE cut-off of >3.3 [22] indicating probable dementia.

Out of 21 analytes measured, 11 had <30% of values below the detection level and were included in the analysis (supplementary material, Appendix S7). Measured in a stable state at enrolment, CCL2 (MCP-1) (13.38 (11.21–17.93) vs. 10.83 (9.03–14.64) *P* = .004) and CXCL2 (MIP-2α) (17.23 (11.80–24.22) vs. 13.49 (10.19–17.98) *P* = .031) were higher in the delirium group compared to the no delirium group (Figure 1 and supplementary material, Appendix S1). GDF15 was non-significantly higher in the delirium group compared to the no delirium group (10.93 (6.81–25.89) vs. 8.06 (0.85–17.00) *P* = .092). ROC analysis for delirium prediction gave an area under the curve (AUC) of 0.67 (95% CI: 0.56–0.78) for CCL2 and 0.63 (95% CI: 0.52–0.74) for CXCL2 (supplementary material, Appendix S6). When participants with and without dementia were compared there was no difference in CCL2 or CXCL2. When groups were stratified by dementia status, the associations of CCL2 with delirium are maintained in both those with and without comorbid dementia. CXCL2 was only significantly higher in delirium in the subgroup without dementia (supplementary material, Appendix S8).

**Figure 1:**
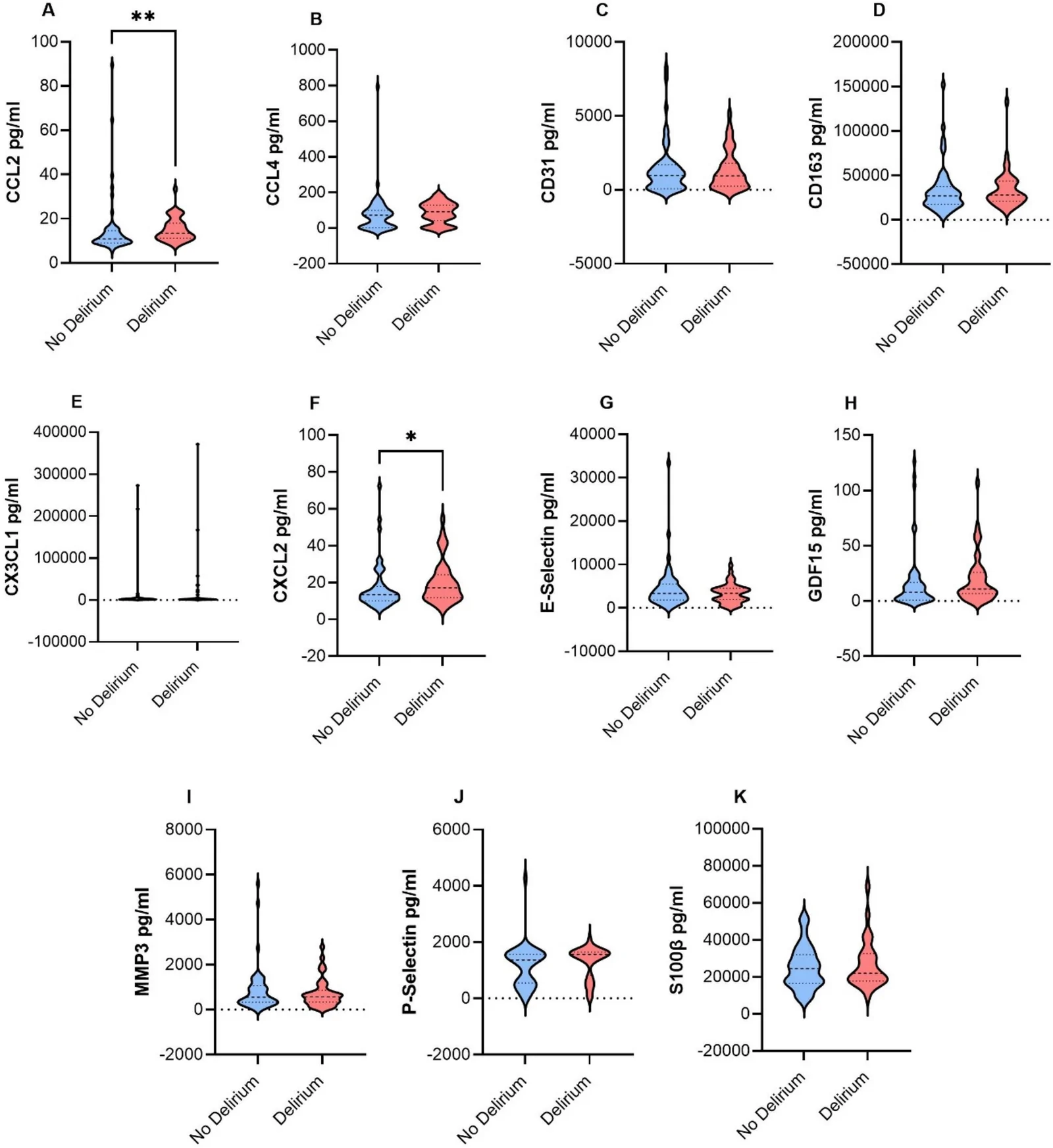
(A–K) Represent the median and IQR of measured analytes in participants who had no episodes of delirium vs. participants who had ≥1 episodes of delirium in the 2-year follow up period. Analytes were included in the figure if >70% of participants had results above the detection limit. Groups were compared using Mann–Whitney U test and significance was set at 95%. *=*P*<0.05, **=*P*<0.001.

## Discussion

In this community-based population of older people with frailty, delirium was common. CCL2 and CXCL2 were higher in those who developed delirium. Stratified analysis revealed this difference remained in those without comorbid dementia.

### Immune cell migration, immune ageing and delirium vulnerability

CCL2 and CXCL2 are chemokines that attract immune cells to sites of inflammation. Both are also Senescence-Associated Secretory Phenotype (SASP) [7] components. As the groups were matched for age, raised levels of these chemokines support the hypothesis that accelerated immune ageing and immune cell migration, contribute to delirium vulnerability. Furthermore, that these chemokine differences are seen in the subgroup without comorbid dementia suggests this may be an important mechanism for delirium vulnerability in those with more resilient brains.

Previous studies support our findings, demonstrating higher levels of CCL2 at baseline in those that developed delirium [8, 9]. Furthermore, an animal model of delirium demonstrated increased brain infiltration of monocytes expressing the CCL2 receptor (CCR2), with resolution of symptoms if CCR2^+^ monocyte recruitment was blocked [10]. CCL2 is also raised in mild cognitive impairment (MCI) and Alzheimer’s Disease [11]. That CCL2 was raised in delirium in the subgroup with dementia, suggests this chemokine could explain the mechanism for higher rates of delirium in dementia. However, we found no difference in CCL2 between those with and without dementia and this relationship should be examined further in larger sample groups.

In contrast, the role of CXCL2 in delirium pathophysiology has not been investigated in humans, with only one study in a mouse model of delirium showing an upregulation of CXCL2 in the hippocampus and serum [12]. CXCL1 binds to the same receptors as CXCL2 (CXCR1/2), and is upregulated in mouse models of delirium [13] and in ICU delirium [14, 15], although not as a predictive marker of delirium. The CXCL2/CXCR1/2 axis therefore warrants further investigation.

Although higher CCL2 and CXCL2 were associated with delirium development, other pro-inflammatory markers previously associated with delirium were not. In some cases, this was due to a high proportion with undetectable levels. A recent review demonstrated that many studies do not adequately report their approach to undetectable values [16] and this may explain variations in the literature, especially in clinically stable participants. Peripheral levels of cytokines may also not reflect levels within the brain. Future work could use more sensitive techniques such as proteomics.

### Other markers of frailty and immune ageing and delirium vulnerability

We also hypothesised that other markers of frailty and accelerated immune ageing may explain delirium vulnerability. However, none of these markers were significantly raised. It was difficult to detect differences in frailty markers with high frailty prevalence. Or again, there was a high proportion of undetectable cytokines. GDF-15 modulates inflammation, has been associated with frailty and is a SASP component [17]. A large proteome-wide association study identified GDF-15 as the top protein associated with incident delirium [18]. We found higher levels of GDF-15 in delirium, although this did not reach significance. However, it adds to evidence that GDF-15 warrants further investigation as a marker of delirium vulnerability.

### Blood–brain barrier dysfunction and delirium vulnerability

We found no evidence that markers of BBB dysfunction were predictive of delirium. Although studies have found S100β was raised in delirium [19, 20], no studies have found baseline S100β to be predictive of delirium. These markers are good surrogates for direct BBB dysfunction, however, can be raised in other inflammatory states. We did not measure GFAP, a newer marker which predicts post-operative delirium [21]. Future research should investigate if this is predictive in a community population.

### Strengths and limitations

This study has several strengths. First, a high proportion of participants were living with frailty, a group at greatest risk of delirium but often excluded from research, making the findings relevant to the most vulnerable. Second, delirium incidence was high. Third, delirium detection was strong as diagnoses were made by research staff, rather than relying on hospital records. There were, however, some limitations. First, the small sample limits generalisability. Second, multiple analytes were compared between groups; however, as the study was small and exploratory, we elected not to adjust for multiple comparisons. This increases the risk of type I statistical errors. Third, although delirium capture was good, cases may have been missed. Fourth, the interval between the baseline bloods and the delirium episode, and the number of episodes, was variable. Finally, high mortality led to variable follow up time.

### Future directions

This study found the chemokines CCL2 and CXCL2 defined delirium vulnerability in this population. Future work should study chemokine gradients between the CSF and periphery and elucidate the role of immune cell recruitment and brain infiltration in delirium to identify potential treatment targets. CCL2 and CXCL2 are also markers of accelerated immune ageing [7] and the development of geroprotective agents that target this, may be protective for delirium.

## Supplementary Material

Supplementary_materials_afag290

## Data Availability

The data that support the findings of this study are available from the corresponding author upon reasonable request.
