# Supplementary material for: CCL2 and CXCL2 are markers of delirium vulnerability in community dwelling older people
Source: Age Ageing. 2026 Sep 30;55(9):afag290. doi: 10.1093/ageing/afag290 (PMC13625644; doi:10.1093/ageing/afag290)
Supplement: Supplementary file 1 [file supplementary_materials_afag290.docx]

CCL2 and CXCL2 are Markers of Delirium Vulnerability in Community Dwelling Older People - Supplementary Material

[Appendix 1	1](#_Toc239710649)

[Appendix 2	3](#_Toc239710650)

[Appendix 3	4](#_Toc239710651)

[Appendix 4	6](#_Toc239710652)

[Appendix 5	9](#_Toc239710653)

[Appendix 6	10](#_Toc239710654)

[Appendix 7	11](#_Toc239710655)

[Appendix 8	12](#_Toc239710656)

## Appendix 1

**Table 1** Comparison of analytes included in the multiplex assay between participants that had ≥1 episode of delirium and those that had no episodes

| Multiplex Assay Analyte | No Episodes of Delirium  (n=55) | One or More Episode of Delirium (n=43) | Significance |
| --- | --- | --- | --- |
| CCL2 (MCP-1)  (pg/ml) | 10.83 (9.03-14.64) | 13.38 (11.21-17.93) | **p=0.004** |
| CD31 (PECAM-1)  (pg/ml) | 971.77 (74.68-1690.00) | 943.82 (244.5-1806.00) | p=0.931 |
| CX3CL1 (Fractalkine)  (pg/ml) | 2166.09 (1374.00-3688.00) | 2188.25 (1404.00-4052.00) | p=0.624 |
| CXCL2 (MIP2-α)  (pg/ml) | 13.49 (10.19-17.98) | 17.23 (11.80-24.22) | **p=0.031** |
| MMP3  (pg/ml) | 556.08 (325.80-1062.00) | 562.29 (340.20-865.20) | p=0.753 |
| E-Selectin  (pg/ml) | 3364.26 (1835.00-5536.00) | 3413.99 (1922.00-4535.00) | p=0.429 |
| CCL4 (MIP1β)  (pg/ml) | 72.00 (1.61-99.65) | 91.07 (42.12-128.90) | p=0.127 |
| CD163  (pg/ml) | 26875.16 (17401.00-37420.00) | 27717.87 (20925.00-43338.00) | p=0.346 |
| GDF15 (MIC-1)  (pg/ml) | 8.06 (0.85-17.00) | 10.93 (6.81-25.89) | p=0.092 |
| P-Selectin*  (pg/ml) | 1361.00 (550.50-1564.00) | 1565.00 (821.00-1640.00) | p=0.081 |
| S100β**  (pg/ml) | 24451.19 (16588.00-32092.00) | 21922.83 (17745.00-32633.00) | p=0.640 |

Analytes were included in the table if >70% of participants had results above the detection limit. Displayed values are medians and interquartile ranges (IQR).Mann-Whitney U test was used to compare groups. Statistical significance was set at 95%. *Samples size for p-selectin is n=40 ≥1 episodes of delirium and n=55 no episodes of delirium. **Sample size for S100B is n=28 ≥1 episodes of delirium and n=55 no episodes of delirium.

| Demographics | Study Sample  (n=98) | Total Population  (n=210) |
| --- | --- | --- |
| One or more episode of Delirium (%) | 43 (43.9%) | 89 (42.4%) |
| Age (years, median/IQR) | 88.0 (81.8-92.0) | 86.0 (78.0-91.0) |
| Female (%) | 64 (65.3%) | 138 (65.7%) |
| Frailty Index Score (mean/SD) | 0.38 (0.11) | 0.39 (0.13) |
| Frail (%)* | 89 (90.8%) | 185 (88.1%) |
| IQCODE >3.3 (%) | 54 (55.1%) | 121 (57.6%) |
| IQCODE (median/IQR) | 3.38 (3.06-4.07) | 3.38 (3.06-4.00) |
| Barthel Index (median/IQR) | 17 (15-18) | 17 (14-18) |
| Totally Dependent (%) | 90 (91.8%) | 189 (90.0%) |
| Age Adjusted CCI  (Median/IQR) | 6 (5-8) | 6 (5-8) |
| Education (Years, median/IQR) | 8 (7-12) | 9 (7-12) |
| Adjusted MOCA (median/IQR) | 18 (14-22) | 18 (14-22) |
| Mortality within study period (%) | 29 (29.6%) | 63 (30.0%) |

**Table 2** Comparison of demographics between the sample included in this paper and the full original study sample

## Appendix 2

*SD – Standard Deviation, IQR – Interquartile Range, CCI – Charlson Comorbidity Index, IQCODE – Informant Questionnaire on Cognitive Decline in the Elderly, MOCA – Montreal Cognitive Assessment
*FI Cut off Value ≥0.25*

| **Immune Ageing, Senescence and Frailty** | |
| --- | --- |
| IL-6 | Pro-inflammatory cytokine. Raised in inflammaging. |
| IL-10 | Anti-inflammatory cytokine. Involved in inflammaging. |
| CCL4/MIP1β | Involved in the recruitment and activation of cells expressing CCR2 or CCR5 including granulocytes, t lymphocytes and NK cells. Indicated as a marker for frailty/sarcopenia. |
| MIF | Regulates macrophages through suppressing the anti-inflammatory effects of glucocorticoids. Indicated as a marker for frailty/sarcopenia. |
| CXCL9/MIG | Released by several cells in response to IFN-γ. Particularly important in the chemoattraction of T-lymphocytes of the TH-1 phenotype. Marker of epigenetic age. |
| GDF-15/MIC-1 | Member of the TGFβ superfamily. Indicated as a marker of frailty/sarcopenia. |
| Osteopontin/OPN | Matricellular protein that mediates diverse biological functions. Indicated as a marker of frailty/sarcopenia. |
| CXCL1 | Chemoattractant for a number of cells, especially neutrophils. Marker of immune ageing. |
| **Monocyte activation, migration, adhesion and activity** | |
| IL-8 | Chemokine produced by macrophages that leads to neutrophil recruitment. |
| CCL2/MCP-1 | Chemokine that attracts monocyte to site of inflammation. |
| CX3CL1/Fractalkine | Soluble CX3CL1 potently chemoattracts T cells and monocytes. Essential for microglial migration. |
| e-selectin | Cell adhesion molecule important in recruitment of leukocytes. |
| p-selectin | Cell adhesion molecule important in recruitment of leukocytes. |
| CD31/PECAM-1 | Cell adhesion molecule important in monocyte transmigration. Raised levels in the blood may indicated BBB breakdown. |
| CXCL2/MIP2-α | Secreted by monocytes/macrophages and attracts neutrophils. |
| GM-CSF | Promotes proliferation of granulocytes and monocytes. |
| CD163 | Marker of cells of the monocyte/macrophage lineage. Soluble form upregulated in several inflammatory conditions. |
| **Blood Brain Barrier (BBB) dysfunction** | |
| S100β | Released by astrocytes. Levels in the blood stream increase following neuronal injury and has been used as a marker of BBB permeability. |
| NSE | Glycolytic enzyme found in neurones. Levels rise following neuronal injury and levels in the blood have been used as a marker of BBB permability. |
| MMP-3 | Matrix metalloprotein that is involved in the breakdown of the extracellular matrix. Has been shown to increase BBB permability. |
| **Identified as possible biomarker for risk of delirium from literature review** | |
| IL2 | Secreted by CD4+ and CD8+ T cells and promotes T cell differentiation and enhances the killing abilities of NK cells and CD8+ T cells. Evidence of higher level in those at risk of delirium. |

## Appendix 3

**Figure 1** Markers included in the Multiplex Panel and Rationale for Inclusion

## Appendix 4

### Methods Annexe

#### Study Design and Participants

Participants were recruited between May 2015-July 2016. Exclusion criteria were: life expectancy <1 week, Lewy body dementia, psychiatric illness (except dementia), or for whom substance misuse was the main indication for domiciliary services.

#### Clinical Assessments

Once per week the domiciliary care team were asked the Single Question in Delirium. If there was any suspicion of delirium or the participant had been hospitalised for any reason the project doctor was alerted within 24 hours. The participant was then assessed daily for delirium by the study doctor, two study nurses or a neurologist trained in delirium assessment. Delirium was diagnosed using Diagnostic and Statistical Manual of Mental Disorders, fifth edition criteria through a test if attention, a test of orientation with the Abbreviated Mental Test 4, and a test of arousal with the Richmond Agitation-Sedation Scale and the Observational Scale for Level of Arousal.

The CASCADE research team undertook baseline assessments. Assessments included the Montreal Cognitive Assessments (MoCA), Informant Questionnaire on Cognitive Decline in the Elderly (IQCODE), age-adjusted Charlson Comorbidity Index (CCI), Barthel Index and a Frailty index at baseline. The frailty index used 34 domains and was calculated by dividing the number of domains were the participant had a problem by the number of domains assessed. Although the original study used 48 domains the index has been shown to be valid if at least 30 domains were measured. A Frailty Index cut off of ≥0.25 was used to define frailty. Age-adjust CCI involved adding a further point for every decade of life past 50 to the comorbidity score. A consensus dementia diagnosis was made using all available baseline information and according to International Classification of Disease 10 criteria independently by a junior doctor and a psychiatrist. Disagreements were discussed until a consensus was reached.

Baseline blood samples were taken using BD Vacutainer SST II Advance Tubes 8·5 mL (Becton Dickinson, Plymouth, UK)

#### Laboratory Methods

Baseline whole blood samples were stored at room temperature for 30–120 min. Samples were then centrifuged for 15 min at a fixed angle at 3500 revolutions per min and the supernatant (serum) aliquoted into 1ml volumes. Samples were stored temporarily at -32°C before being moved to long-term storage at -80°C. Samples were shipped from Norway to the UK in November 2022. Temperature was monitored throughout and maintained at -80°C.

Multiplex analysis was performed using a Human Luminex Discovery Assay Kit (R&D Systems, USA).  Reagents were prepared as per manufacturer instructions. Serum samples were thawed at room temperature. Standard or serum was added to each well in a 96 well plate. The magnetic bead solution was then added to each well and incubated for 2 hours. The plate was washed using the Bio-Plex Pro Wash Station with wash buffer three times. The biotinylated antibody solution was then added to each well and incubated for one hour. The plate was then washed using the method above. Streptavidin-PE was then added to each well and incubated for 30. The same wash procedure was then repeated. The plate was then read on the Bio-Plex 200 analyser (Bio-Rad, California, US).

#### Data Analysis

If ≥30% of values for a given marker were below the detectable limit no further analysis were performed. If <30% of values were below the detachable limit, values below the detectable limit were assigned the value of half of the lowest recorded value.

## Appendix 5

**Table 3** Domains Included in the Frailty Index

**Frailty Index Domain**

Coronary Heart Disease

Atrial Fibrillation

Heart Failure

Diabetes

Hypertension

Peripheral Vascular Disease

Stroke/TIA

Anxiety Neurotic Disorder

Rheumatic Disease

Asthma

Malignancy

Kidney Disease

COPD

Any Depression

Sensory Impairment

Polypharmacy

Assistance Eating

Assistance Dressing

Assistance Personal Care

Assistance Mobility Transfers

Assistance Transfers

Assistance Showering

Assistance Toileting

Assistance Shopping

Assistance Housework

Assistance Managing Finances

Can Climb Stairs

Assistance Heavy Housework

Assistance Medication Management

MoCA

Gait Speed

Grip Strength

BMI

Weight Loss

Appendix 6

**Figure 2** Receiver operating characterisation curve (ROC) for prediction of delirium based on the serum levels of CCL2 and CXLC2 measured by multiplex assay. and delirium CCL2 AUC=0.67 (95% CI 0.56-0.78), CXCL2 AUC= 0.63 (95% CI 0.52-0.74)

## Appendix 7

**Table 4** Percentage of Participants with Results Above the Level of Detection for Each Analyte in the Panel

| **Analyte** | **Percentage of Participants with Results Above the Detection Limit** |
| --- | --- |
| **CCL2** | 100% |
| **CD31** | 100% |
| **CX3CL1** | 100% |
| **CXCL2** | 100% |
| **NSE** | 8.2% |
| **GM-CSF** | 7.1% |
| **IL-6** | 24.5% |
| **IL-10** | 60.2% |
| **MMP-3** | 100% |
| **E-selectin** | 100% |
| **CCL4** | 72.4% |
| **CD163** | 98% |
| **CXCL1** | 2% |
| **CXCL9** | 18.4% |
| **GDF-15** | 80.6% |
| **IL-2** | 5.1% |
| **IL-8** | 59.2% |
| **MIF** | 19.4% |
| **Osteopontin** | 59.2% |
| **P-selectin** | 95.9% |
| **S100β** | 100% |

n=98. Analytes shaded in red had <70% of participants with results below the detection limit and were therefore not included in the analysis.

## Appendix 8

**Table 5** Comparison of CCL2 and CXCL2 between participants that had ≥1 episode of delirium and those that had no episodes, stratified by dementia status

| **Multiplex Assay Analyte** | **Dementia**  **(n=50)** | | | **No Dementia**  **(n=48)** | | | **Significance** |
| --- | --- | --- | --- | --- | --- | --- | --- |
|  | **Delirium (n=30)** | **No Delirium (n=20)** | **Significance** | **Delirium (n=13)** | **No Delirium (n=35)** | **Significance** |  |
| **CCL2 (pg/ml)** | 13.01 (11.05-15.95) | 10.35 (8.75-14.70) | **p=0.021** | 17.66 (11.89-21.09) | 11.77 (9.20-14.64) | **p=0.022** |  |
|  | 11.81 9.98-15.59) | | | 12.32 (9.63-17.28) | | | p=0.662 |
| **CXCL2**  **(pg/ml)** | 15.57 (11.58-22.52) | 12.66 (10.49-15.95) | p=0.166 | 20.56 (14.69-35.54) | 17.66 (11.88-21.09) | **p=0.035** |  |
|  | 13.86 (10.91-26.80) | | | 14.09 (10.72-20.65? | | | p=0.634 |
